# Self-Reported Treatment for Hearing Loss in Adults Living With or Without Human Immunodeficiency Virus (HIV)

**DOI:** 10.7759/cureus.103539

**Published:** 2026-02-13

**Authors:** Peter Torre III, Deanna Ware, Chukwuemeka N Okafor, Gayle Springer, Howard Hoffman, Christopher Cox, Michael Plankey

**Affiliations:** 1 School of Speech, Language, and Hearing Sciences, San Diego State University, San Diego, USA; 2 Department of Medicine, Georgetown University Medical Center, Washington, DC, USA; 3 Department of Medicine, Division of Infectious Diseases, Long School of Medicine, University of Texas Health Science Center at San Antonio, San Antonio, USA; 4 Department of Epidemiology, Johns Hopkins University Bloomberg School of Public Health, Baltimore, USA; 5 Department of Epidemiology, National Institute on Deafness and Other Communication Disorders, National Institutes of Health, Bethesda, USA

**Keywords:** hearing loss, hearing treatment, hiv, macs/wihs combined cohort study, self-report

## Abstract

Human immunodeficiency virus (HIV) and its treatment have been associated with hearing loss. For those who can benefit from treatment for hearing loss (i.e., hearing aids), uptake for services is low, likely due to stigma. There is also stigma for people living with HIV (PWH). As such, it is not known how both hearing loss and HIV status affect whether or not adults seek treatment for hearing loss. Therefore, the purpose of this cross-sectional (PT1) study was to evaluate self-reported hearing ability and hearing loss treatment in middle-aged and aged PWH and people without HIV (PWOH). Participants in the Multicenter AIDS Cohort Study (MACS) Women's Interagency HIV Study (WIHS) Clinical Cohort Study (MWCCS) were asked questions related to their hearing ability and whether they sought hearing services. Among the 933 participants who reported hearing problems, 143 (15.3%) reported using hearing amplification; there was no difference by HIV serostatus. Almost 50% of participants who reported having a lot of trouble hearing did not report using any hearing amplification. Increased public education about hearing loss and amplification is needed, and this should come from audiologists and primary care physicians.

## Introduction

The prevalence of hearing loss in older US adults (71 years or older) is approximately 65% [1]. It is projected that the number of older US adults with hearing loss will be >73 million by 2060 [2]. Hearing loss is associated with poorer physical function [3], but also, older adults with untreated hearing loss experience greater healthcare costs and higher healthcare utilization than those with normal hearing [4]. However, between 20% [5] and 30% [1] of older adults with hearing loss report using a hearing aid. These estimates are lower in Black and Hispanic older adults and low-income individuals [1,6].

The process of acquiring hearing aids is a complex one; individuals are aware of hearing problems for approximately 10 years before acting [7]. Greater self-reported hearing problems are a significant predictor of entering a hearing aid evaluation period [8] and hearing aid adaptation [9-11]. There are non-audiological barriers to hearing aid uptake that extend beyond the cost; perceived benefit, personality traits, and locus of control (a psychological concept describing how much people believe their own actions influence the outcomes and events in their lives) are the complex factors affecting hearing aid utilization [12].

Human immunodeficiency virus (HIV) disproportionally affects those who are socially and economically marginalized, and those with the highest social vulnerability may endure barriers to HIV care services because of housing insecurity, limited access to transportation, and HIV stigma [13]. Although the gap in life expectancy between people with HIV (PWH) with access to care and people without HIV (PWOH) is down to eight years [14], access to care for PWH is improving; it remains suboptimal [15]. Maintaining access to care, for HIV or general health, is important. Unfortunately, in a longitudinal study of over 12,000 PWH, after 10 years of entry into HIV care, over 20% of PWH were lost to care [16].

Older PWH are at greater risk for hearing loss compared to older PWOH [17]. Moreover, hearing loss affects one's ability to perceive auditory events and communication with others, which can contribute to social isolation leading to a decreased quality of life [18], conditions that are also prevalent among PWH. Given the rate of PWH lost to follow-up in HIV care and the delay in acquiring hearing intervention, it is important to examine whether or not PWH seek hearing health services. As a result, the purpose of this study was to evaluate self-reported hearing ability and hearing loss treatment in PWH and PWOH.

## Materials and methods

The Multicenter AIDS Cohort Study (MACS), which began in 1984, and the Women's Interagency HIV Study (WIHS), which began in 1994, were combined in 2019 to become the MACS/WIHS Combined Cohort Study (MWCCS) [19]. MWCCS participants attend annual visits for standardized clinical examinations, specimen collection, and self-reporting of sociodemographic, behavioral, and medical data. Hearing-specific data were obtained at a separate visit and included participants who responded to interviewer-administered questions related to their hearing ability between October 1, 2020, and September 30, 2022. Given that individuals who report excellent hearing are unlikely to seek hearing services, participants who reported a little trouble hearing or worse were included in this study.

Measures

Participants were asked two questions specific to their hearing from the 2019-2020 National Health and Nutrition Examination Survey (NHANES) hearing questionnaire [20]. First, they were asked: "Is your hearing 'excellent', 'good', 'a little trouble hearing', 'moderate trouble hearing', or 'a lot of trouble hearing'?" Participants with at least a little trouble hearing were then asked: "Have you used any type of device to amplify sounds (i.e., hearing aid, assistive listening device) to treat hearing impairment?" (Hearing amplification: yes, no).

Covariates

Covariates included age (years), HIV serostatus (yes, no), assigned sex at birth (female, male), race/ethnicity (White non-Hispanic, Black non-Hispanic, Hispanic, other), health insurance coverage (none, private, Medicare, Medicaid, Veterans Affairs (VA), other/unspecified), and doctor visits within six months (yes, no).

Statistical methods

Sample characteristics, by hearing amplification status, were described using median/interquartile range (IQR) and frequency/percentage, as appropriate. The differences in the distributions of sample characteristics were tested using the chi-squared, Fisher's exact (for cell sizes <10%), and Wilcoxon (for continuous variables) tests. Stacked bar charts were generated by HIV status to visualize the distributions of hearing amplification status by hearing rating. P-values less than 0.05 were considered statistically significant. All analyses were performed in SAS 9.4 (SAS Institute Inc., Cary, NC, USA).

## Results

There were 3704 who answered the hearing-related questions, and 933 (25.2%) reported a little trouble hearing or worse. Among those 933 participants who reported hearing problems and were included in the analysis, 790 (40.9% PWOH, 59.1% PWH) of whom reported no hearing amplification (84.7%) (Table 1). Those who did not report hearing amplification were significantly younger (median age 60 years vs 69 years) than those who did report hearing amplification. A higher percentage of PWH did not report hearing amplification compared to PWOH, and the percentages were similar between PWH and PWOH who did report hearing amplification; this difference, however, was not statistically significant. Additional demographic variables (e.g., sex, race/ethnicity, insurance, and medical visits) are also shown in Table 1.

**Table 1:** Analytic sample characteristics stratified by hearing treatment status ^1^Wilcoxon two-sample test. ^2^Fisher's exact test due to at least one cell <5. ^3^75 participants had missing data. IQR: interquartile range; PWOH: people without human immunodeficiency virus; PWH: people with human immunodeficiency virus

	Overall (N=933)		Test statistic
	No hearing amplification (N=790)	Hearing amplification (N=143)	
Age in years, median (IQR)^1^	60 (52-66)	69 (60-76)	Z=8.39; p<0.001
HIV status			
PWOH	323 (40.9%)	70 (49.0%)	χ^2^(1; N=933)=3.23; p=0.07; f=-0.06
PWH	467 (59.1%)	73 (51.1%)	
Sex			
Female	413 (52.3%)	42 (29.4%)	χ^2^(1; N=933)=25.43; p<0.01; f=0.17
Male	377 (47.7%)	101 (70.6%)	
Race/ethnicity^2^			
White non-Hispanic	267 (33.8%)	92 (64.3%)	p<0.01
Black non-Hispanic	387 (49.0%)	34 (24.8%)	
Hispanic	106 (13.4%)	15 (10.5%)	
Other	30 (3.8%)	2 (1.4%)	
Insurance coverage^2,3^			
None	50 (6.7%)	1 (0.8%)	p<0.01
Private	135 (18.5%)	15 (11.5%)	
Medicaid	198 (27.2%)	15 (11.5%)	
Medicare	282 (38.7%)	83 (63.9%)	
Other	15 (2.1%)	10 (7.7%)	
Non-specified	48 (6.6%)	6 (4.6%)	
Doctor visit since last visit (6 months)^3^			
No	74 (10.2%)	79 (6.9%)	χ^2^(1; N=858)=1.33; p=0.25; f=0.04
Yes	654 (89.8%)	121 (93.8%)	

**Figure 1:**
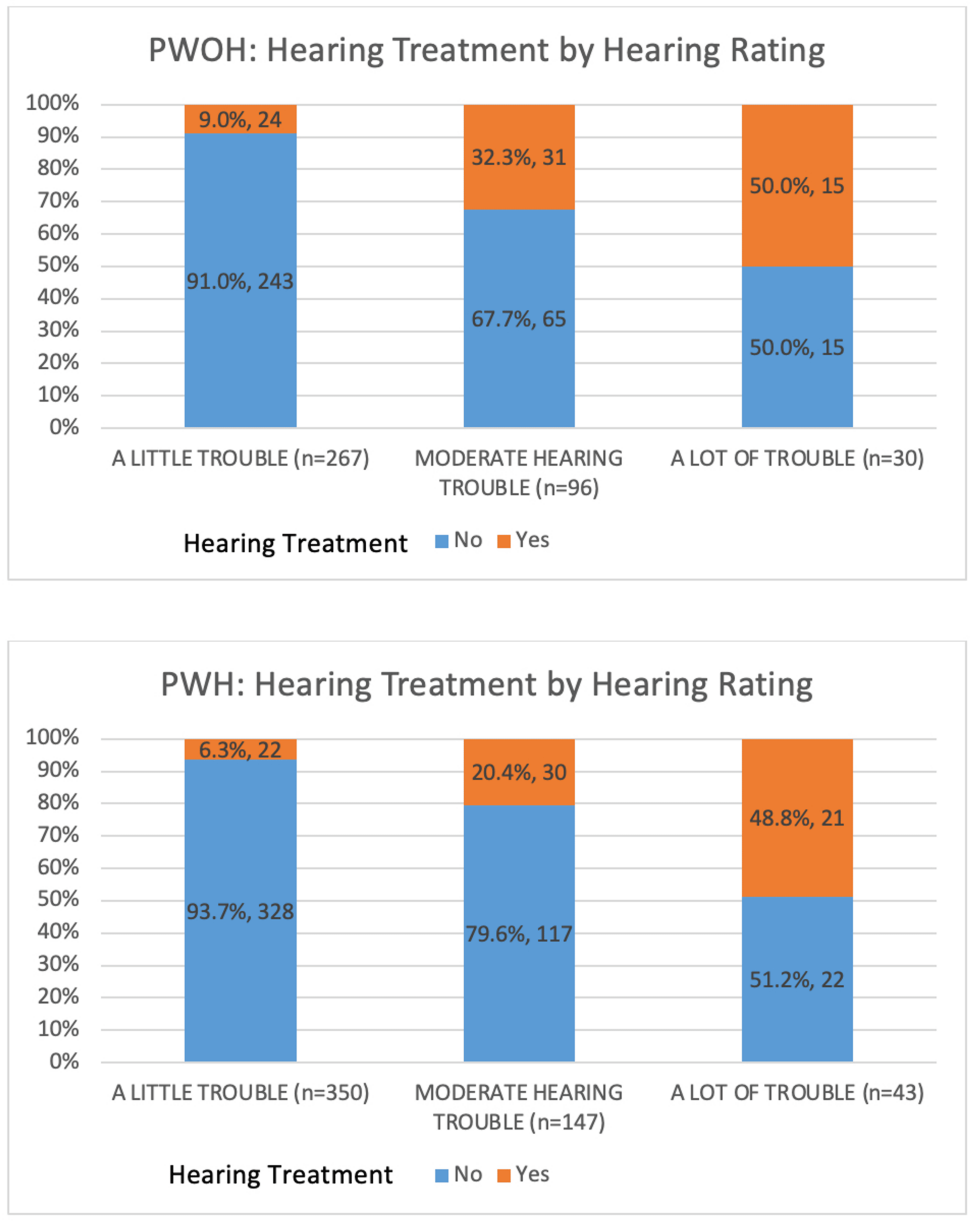
Hearing treatment status by hearing rating, stratified by HIV serostatus HIV: human immunodeficiency virus; PWOH: people without HIV; PWH: people with HIV

For PWOH, 24 (9.0%), 31 (32.3%), and 15 (50.0%) participants who reported a little trouble, moderate trouble, and a lot of trouble hearing, respectively, used hearing amplification. Among PWH, 22 (6.3%), 30 (20.4%), and 21 (48.8%) participants who reported a little trouble, moderate trouble, and a lot of trouble hearing, respectively, reported using hearing amplification (Figure 1).

## Discussion

Almost 85% (790 of 933) of those who reported a little trouble or worse hearing did not report using any hearing amplification. For the 15% (N=143) who did report some type of amplification, this percentage is lower than recent data for those who specifically use hearing aids [1]. Those who did report using hearing amplification were significantly older and male, yet there was no unadjusted association observed between HIV serostatus who reported using hearing amplification and those who did not. As participants reported greater difficulty hearing, there was an increase in reported use of amplification, which is consistent with previous research [9-11]. A somewhat concerning outcome of the current data, however, is that approximately 50% of participants who reported having a lot of trouble hearing still did not report using any hearing amplification.

It is not known what barriers exist within the current study for half of the participants not seeking hearing amplification even for MWCCS participants who are engaged in care. For PWH, it is possible that other comorbidities (e.g., hypertension, hyperlipidemia) [21] take priority in PWH who have hearing problems. And with the availability of over-the-counter (OTC) hearing aids, only 2% (N=25) of adults who reported hearing difficulty have purchased OTC hearing aids, and only 4% (N=48) reported that they are likely to purchase OTC hearing aids within a year [22]. It is possible, however, that hearing aid uptake rates may be different for adults enrolled in the MWCCS. Social value is the self-perceived benefit of a hearing aid combined with the benefit perceived by others. Those who report greater hearing problems likely experience more communication difficulty in social situations and, as a result, are more socially motivated to purchase hearing aids. Further, answering yes to "Do friends and relatives think you have a hearing problem?" was significantly associated with hearing aid adoption [23], which contributes to the social value of hearing aids, yet still does not account for many who do not utilize hearing aids. Further, age-related stigma associated with hearing aids which can lead to feelings of shame and embarrassment [24] along with the diversity of listening environments often requires support for those seeking hearing aids.

There are some limitations with the current research. First, no diagnostic audiometric testing was completed in order to assess hearing sensitivity; self-reported hearing ability was used. Older adults, however, are accurate when assessing their hearing ability via self-report; in fact, some overestimate hearing loss [25]. Given the number of participants in the current study, self-reported hearing was the most efficient method. Second, it is possible that for the hearing amplification question, participants may have interpreted as ever used amplification, and as a result, current hearing amplification use may not have fully been assessed. 

## Conclusions

There was no unadjusted difference by HIV serostatus observed in the current study, and there was low hearing amplification uptake. The reason for the lack of hearing aid utilization is likely an interaction of many factors. As mentioned previously, those who need hearing aids struggle with stigma, feelings of shame and embarrassment, and difficulty in listening environments and often require guidance on how to seek aural rehabilitation. Professional recommendations are a strong motivator where audiologists can clearly communicate the importance of professional care. Audiologists can emphasize amplification as a mechanism for empowerment, enabling improved communication in all settings, meaningful connections with family, and more active participation in social and recreational activities. Increased public education about hearing loss and amplification options will also minimize the negative opinion of hearing aids, and this can come from both audiologists and primary care physicians.
